# 4′-Hydroxydehydrokawain Mitigate the Cytotoxicity of Citrinin in Porcine Intestinal Epithelial Cells

**DOI:** 10.3390/toxics13040315

**Published:** 2025-04-18

**Authors:** Seung Joon Lim, Sangsu Shin, Sang In Lee

**Affiliations:** 1 Department of Animal Science and Biotechnology, Kyungpook National University, Sangju-si 37224, Republic of Korea; coocu1214@knu.ac.kr (S.J.L.); sss@knu.ac.kr (S.S.); 2 Research Institute for Innovative Animal Science, Department of Animal Science and Biotechnology, Kyungpook National University, Sangju-si 37224, Republic of Korea

**Keywords:** citrinin, cell cycle arrest, apoptosis, TGF-beta signaling, 4′-hydroxydehydrokawain, IPEC-J2 cells

## Abstract

Citrinin (CTN) is a mycotoxin that adversely affects livestock by contaminating stored grains, leading to significant health and economic impacts. This study investigates the toxicological effects of CTN on porcine small intestinal epithelial cells (IPEC-J2) and explores potential mitigation strategies using natural products and chemical inhibitors. Our study demonstrates that CTN induces cytotoxicity through the TGF-β signaling pathway, triggering apoptosis and G2/M phase cell cycle arrest. We examined cell viability, cell cycle progression, and gene expression changes in IPEC-J2 cells treated with CTN, 4′-Hydroxydehydrokawain (4-HDK), and LY-364947, a TGF-β receptor inhibitor. LY-364947 treatment confirmed that CTN-induced toxicity is mediated through TGF-β signaling. Although 4-HDK alleviated CTN-induced cytotoxicity by improving cell viability and reducing apoptosis, its direct involvement in TGF-β inhibition remains unclear. These results suggest that CTN disrupts intestinal epithelial cell homeostasis via TGF-β activation, whereas 4-HDK may exert protective effects through an alternative mechanism. Our study provides novel insights into CTN-induced toxicity mechanisms and highlights the therapeutic potential of 4-HDK as a mitigator of mycotoxin-induced cellular damage.

## 1. Introduction

Mycotoxins, toxic secondary metabolites produced by fungi, can cause health problems in humans and farm animals [1]. Exposure to mycotoxins can have direct adverse effects on animals, impacting their health and productivity while also posing risks to humans through the consumption of contaminated animal-derived products, ultimately leading to economic losses [2]. Mycotoxins, including aflatoxin B1, fumonisin B1, deoxynivalenol (DON), ochratoxin A, and citrinin (CTN), have toxic effects on animal cells [3,4,5,6,7]. Mycotoxins damage cells by causing oxidative stress, membrane disruption, apoptosis, and metabolic impairment, leading to intestinal barrier dysfunction [8,9]. Citrinin is produced by various fungal species, including members of the genera Aspergillus, Penicillium, and Monascus [10]. It is primarily found in stored grains but has also been detected in beans, fruits, and dairy products [11]. Studies have shown that CTN can induce nephrotoxicity, hepato-toxicity, and genotoxicity [12,13,14]. However, there is currently a lack of research on CTN-induced molecular mechanisms in the pig intestinal epithelium.

The intestinal epithelium forms a major barrier separating our body from the external environment [15]. These intestinal epithelial cells (IECs) form a continuous single-layer sheet at the surface of this barrier, acting as a mediator between the internal and external environments [16]. An important function of this layer is to maintain the integrity of the barrier, limiting the passage of hazardous substances while allowing the passage of certain ions, nutrients, and water [17]. To maintain intestinal integrity, IECs undergo continuous, rapid cell regeneration [18]. Enterocytes, which make up the bulk of IECs, are renewed approximately every 3–5 days through a dynamic equilibrium between cell proliferation in the crypt and cell shedding at the tip of the villus [19,20]. Prolonged activation of specific tumor suppressors, such as the transcription factors p53 and p21, can inhibit cell proliferation in response to DNA damage, which can be triggered by factors such as the unnatural conditions of tissue culture or genetic disorders associated with tumorigenesis and genotoxicity [21]. It is known that cell cycle arrest is caused by various mycotoxins, with detrimental effects on intestinal integrity [22,23,24].

Compound 4′-hydroxydehydrocayne (4-HDK) is one of the eight extracts isolated from the seeds of the Alpinia katsumadai Hayata (AKH) plant in the ginger family, including 7-bis(4-hydroxyphenyl)-3-hydroxy-1,3-heptadien-5-one and seven known compounds: 1,7-bis(4-hydroxyphenyl)-3-hydroxy-1,3,6-heptatrien-5-one, 4′-hydroxydehydrokawain, 5,6-dehydrokawain, 4′,7-dihydroxy-5-methoxyflavanone, cardamomin, helichrysetin, and 4-hydroxybenzaldehyde [25]. A study has shown that AKH possesses antibacterial, antioxidant, anti-inflammatory, and anti-asthmatic properties [26]. Another AKH extract, alpinetin, is known to alleviate ulcerative colitis induced by dextran sulfate sodium in mice [26], as does cardamonin, a natural flavonoid isolated from AKH [27]. AKH includes antibacterial, antioxidant, anti-inflammatory, and anti-asthmatic properties, as well as potential benefits for conditions such as ulcerative colitis [28,29]. However, little research regarding 4-HDK has been published.

Our study focuses on the TGF-β signaling pathway and its role in cell cycle regulation and apoptosis in response to CTN treatment, as well as how HDK and LY-364947 can mitigate these effects. To better understand the molecular mechanisms underlying CTN’s toxicity toward IECs, we assessed cell viability and examined changes in the cell cycle and gene expression patterns following CTN treatment in IPEC-J2 cell-line enterocytes. Additionally, we further investigated the functions of genes that were significantly upregulated or downregulated by CTN.

## 2. Materials and Methods

### 2.1. Cell Culture and Treatment

The IPEC-J2 cell line used in this study was originally derived from the small intestine of a neonatal piglet at Hannover Veterinary School, Germany. It is widely used for in vitro studies of intestinal epithelial function in pigs and has been purchased from Deutsche Sammlung von Mikroorganismen und Zellkulturen (DSMZ) (GmbH, Braunschweig, Germany). Cell line IPEC-J2 enterocyte cells were cultured in a humidified incubator at 37 °C with 5% CO_2_ using Dulbecco's Modified Eagle’s Medium (DMEM) (Thermo Fisher Scientific, Wilmington, DE, USA) supplemented with 10% fetal bovine serum and 1% penicillin–streptomycin. CTN and LY-364947 (both from Sigma–Aldrich, St. Louis, MO, USA) were diluted with dimethyl sulfoxide to treat IPEC-J2 cells.

### 2.2. Cell Viability

The cells were seeded at a density of 5 × 10^3^ per 100 μL in 96-well plates and incubated for 24 h. Following this, the cells were stabilized overnight in fresh media. Then, CTN (Sigma–Aldrich, St. Louis, MO, USA) treatments were performed at concentrations of 100, 120, 140, 160, 180, 200, and 400 μM for 24 h. After 24 h of CTN treatment, the cells were washed with PBS, the media was replaced, and then Water-Soluble Tetrazolium-1 (WST-1) (Roche Diagnostics GmbH, Mannheim, Germany) was added. The cells were incubated for an additional 2 h. The absorbance of the dye was measured using a Glo-Max Discover Multi-Microplate Reader by recording the reading at 450 nm and subtracting the background absorbance at 600 nm.

### 2.3. Gene Expression Profiling

The IPEC-J2 cells used to assess gene expression were seeded at a density of 3 × 10^5^ in 60 mm dishes, incubated overnight in DMEM, and treated with 160 μM CTN. Total RNA was extracted using TRIzol reagent (Thermo Fisher Scientific, Wilmington, DE, USA). The quality and quantity of the RNA were determined using an Agilent 2100 Bioanalyzer with an RNA 6000 Nano Chip (Agilent Technologies, Amstelveen, The Netherlands) and ND-2000 Spectrophotometer (Thermo Fisher Scientific, Waltham, MA, USA). Libraries were constructed using the QuantSeq 3′ mRNA-Seq Library Prep Kit (Lexogen, Vienna, Austria) according to the manufacturer’s instructions for control and treatment RNA. Briefly, 500 ng of total RNA was hybridized to an oligo-dT primer containing an Illumina sequence at its 5′ end, followed by reverse transcription. After RNA template degradation, second-strand synthesis was completed with a mix of random primers containing an Illumina-compatible linker sequence at the 5′ end. Double-strand libraries were purified using magnetic beads and amplified to incorporate necessary adapter sequences for cluster generation. These PCR products were purified to obtain the final libraries. High-throughput single-end 75-cycle sequencing was performed on a NextSeq 500 system (Illumina, Inc., San Diego, CA, USA). Gene expression profiling was performed using Excel-based differential expression analysis and differentially expressed genes (DEGs) were annotated, integrated, and visualized with Gene Ontology (GO) and Kyoto Encyclopedia of Genes and Genomes (KEGG) mappers. DEGs were identified as genes that were up- or downregulated by at least two-fold compared to the control.

### 2.4. Cell Cycle Analysis

Cell cycle analysis was conducted using flow cytometry with PI staining. First, IPEC-J2 cells (1 × 10^5^ cells/mL) were seeded into 35 mm culture dishes and treated with 160 μM CTN for 24 h. After treatment, the cells were harvested using trypsin-ethylenediaminetetraacetic acid (EDTA) and subsequently fixed with 70% ethanol. Following fixation, the cells were washed twice with cold phosphate-buffered saline (PBS), and the supernatant was removed after centrifugation. The cell pellet was then stained with 100 μL of propidium iodide (PI)/RNase Staining Solution (Cell Signaling Technology; Danvers, MA, USA) for 15 min in darkness. The DNA content was analyzed using flow cytometry a FACS Verse flow cytometer (BD Science, San Jose, CA, USA), and the flow cytometry data were analyzed using FlowJo 10.8.1 software (FlowJo, LLC, Ashland, OR, USA).

### 2.5. Annexin-V and Propidium Iodide (PI) Staining

After a 24 h CTN treatment at 160 µM, IPEC-J2 cells were harvested and washed with PBS. Following centrifugation, the supernatants were discarded, and the cells were resuspended in 1× annexin binding buffer. Then, 5 μL of Alexa Fluor 488 Annexin-V (Thermo Fisher Scientific, Wilmington, DE, USA) and 1 μL of a 100 mg/mL PI working solution were added. The cells were incubated at room temperature in the dark for 15 min. Afterward, the cells were stained with DAPI (Vector Laboratories, Burlingame, CA, USA) and mounted on coverslips. Fluorescence microscopy (Korealabtech, Seongnam-si, Gyeonggi-do, Republic of Korea) was used to capture images.

### 2.6. RT–qPCR

Total RNA was extracted from the cells using an AccuPreP Universal RNA Extraction Kit (BioNEER, Daejeon, Korea). The quantity and quality of the RNA were assessed with an Agilent 2100 Bioanalyzer and a Thermo Inc. ND-2000 Spectrophotometer. For cDNA synthesis, 1 μg of total RNA was reverse-transcribed using the DiaStar™ RT Kit (SolGent, Daejeon, Republic of Korea) according to the manufacturer’s instructions. Primers for RT-qPCR were designed using Primer 3 (https://primer3.ut.ee/, accessed on 1 June 2024), and their sequences are listed in Table 1. qPCR was performed using a Taq DNA Polymerase Kit (BioNEER, Daejeon, Republic of Korea) and 20X EvaGreen (Biofact, Daejeon, Republic of Korea). The assays were conducted in triplicate on a Bio-Rad CFX96 Real-Time PCR Detection System with the following thermocycler protocol: initial denaturation at 95 °C for 3 min, followed by 40 cycles of 95 °C for 15 s, 56–57 °C for 15 s, and 72 °C for 15 s. The specificity of amplification was verified by melting curve analysis. Primer efficiencies were calculated using a standard curve generated from serial dilutions of cDNA. Fluorescence data were normalized to the reference gene glyceraldehyde-3-phosphate dehydrogenase (GAPDH), and relative gene expression levels were calculated using the 2−ΔΔCt method. All assays were conducted in compliance with MIQE guidelines to ensure reproducibility and reliability of the results (Table 1).

### 2.7. High-Throughput Screening

For the high-throughput screening analysis, 100 natural products were provided by the National Development Institute for Korean Medicine (Gyeongsan, Republic of Korea) and diluted to a concentration of 1 mg/mL using DMSO. After seeding IPEC-J2 cells at a density of 5 × 10^3^ per 100 μL in 96-well plates for 24 h, they were incubated overnight in media. Subsequently, a set of 100 natural products (NPs) were respectively administered at a concentration of 2 ng/μL along with 160 μM CTN for 24 h. Following a 2-h treatment with WST-1 (Roche Diagnostics GmbH, Mannheim, Germany), cell viability was determined using a microplate reader set to measure the absorbance at 450 nm.

### 2.8. Statistics

All experiments were performed independently with three biological replicates (*n* = 3) for each parameter. Significant differences between treatments were assessed using the general linear model (PROC-GLM) procedure in SAS software, version 9.4 (SAS Institute Inc., Cary, NC, USA). Specifically, cell viability data were analyzed using general linear regression, and gene expression data were analyzed using *t*-tests. Additionally, ANOVA was conducted to evaluate overall differences among groups, followed by Tukey multiple range tests. Significant differences between the treatment and control groups were indicated by the following symbols: * *p* < 0.05, ** *p* < 0.01. Each analysis included three biological replicates per treatment group. A *p*-value < 0.05 was considered significant for all tests.

## 3. Results

### 3.1. Citrinin Decreased the Viability of IPEC-J2 Cells

**Figure 1:**
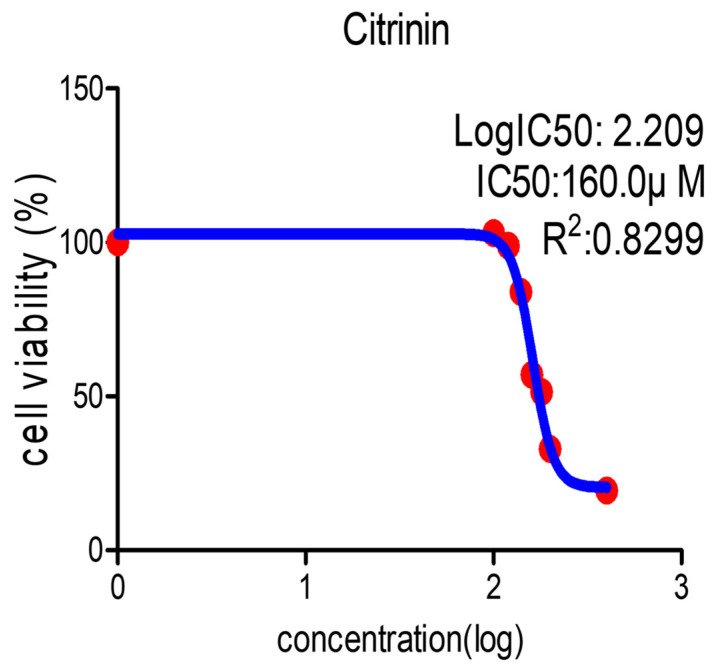
Citrinin decreased viability in IPEC-J2 cells. The cell viability was determined using a WST-1 assay in IPEC-J2 cells incubated in different concentrations of CTN (0, 100, 120, 140, 160, 180, 200, or 400 μM) for 24 h. In the insect, CTN toxicity is expressed by concentration required to inhibit 50% of cell viability (IC50).

To assess the viability of IPEC-J2 cells treated with CTN, we performed a cell proliferation analysis using WST-1 (Figure 1). IPEC-J2 cells were exposed to varying concentrations of CTN: 0, 100, 120, 140, 160, 180, 200, or 400 μM. This decreased in a concentration-dependent manner. In all subsequent experiments, IPEC-J2 cells were treated with CTN (IC50) for 24 h to investigate the underlying molecular mechanisms associated with the toxicity of CTN.

### 3.2. Identification and Validation of DEGs

**Figure 2:**
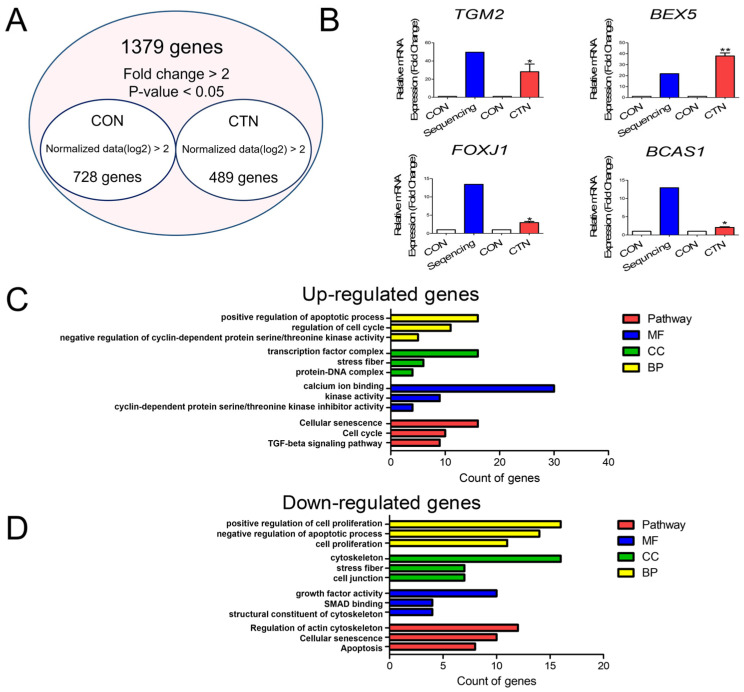
Gene expression profiling was conducted in CTN-treated IPEC-J2 cells. (**A**) Venn diagram illustrating genes whose expression was upregulated or downregulated by at least 2-fold after CTN treatment compared to that of the untreated control (CON). (**B**) The relative expressions of the four most highly upregulated differentially expressed genes (DEGs) in CTN-treated cells assessed through real-time quantitative PCR (*n* = 3). Significant differences between means are indicated by asterisks: * *p* < 0.05 and ** *p* < 0.01. The three most common terms identified in the molecular function (MF), cellular component (CC), and biological processes (BP) categories in a Gene Ontology analysis and in a KEGG pathway enrichment analysis (Pathway) were performed on the upregulated (**C**) and downregulated (**D**) genes.

We conducted gene expression profiling to identify DEGs in small intestinal epithelial cells with or without the 160 μM CTN treatment. Among the 1379 DEGs identified, 782 were upregulated, and 489 were downregulated (Figure 2A). To validate the DEGs, we conducted RT–qPCR analyses to assess the expression of the four genes that showed the highest increase in expression in IPEC-J2 cells treated with CTN, as determined by RNA-seq (Figure 2B). The results revealed that the CTN treatment significantly upgraded TGM2 (*p* < 0.05), BEX5 (*p* < 0.01), FOXJ1 (*p* < 0.05), and BCAS1 (*p* < 0.05) (Figure 2B). A GO analysis of the upregulated DEGs revealed significant terms (Figure 2C). In the “molecular function” category, we found enrichment related to “positive regulation of apoptotic processes”, “regulation of the cell cycle”, and “negative regulation of cyclin-dependent protein serine/threonine kinase activity”. Within the “cellular component” category, terms such as “transcription factor complex”, “stress fiber”, and “protein–DNA complex” were prominent, describing the subcellular localization of these genes. Terms such as “calcium ion binding”, “kinase activity”, and “cyclin-dependent protein serine/threonine kinase inhibitor activity” were associated with the “biological processes” category. According to the KEGG pathway analysis, the major upregulated signaling pathways included “cellular senescence”, “cell cycle”, and the “TGF-beta signaling pathway”. Conversely, the downregulated DEGs (Figure 2D) were linked to terms such as “positive regulation of cell proliferation”, “negative regulation of apoptotic processes”, and “cell proliferation” in the “molecular function” category. Within the “cellular component” category, terms such as “cytoskeleton”, “stress fiber”, and “cell junction” were included. The “biological processes” category included terms such as “growth factor activity”, “SMAD binding”, and “structural constituent of cytoskeleton” to describe the functions of these genes. In the KEGG analysis, the primary downregulated signaling pathways were “Regulation of actin cytoskeleton”, “Cellular senescence”, and “Apoptosis”. Other data are included in the Supplementary Materials.

### 3.3. CTN Induces G2/M Phase Cell Cycle Arrest and Apoptosis in IPEC-J2 Cells

**Figure 3:**
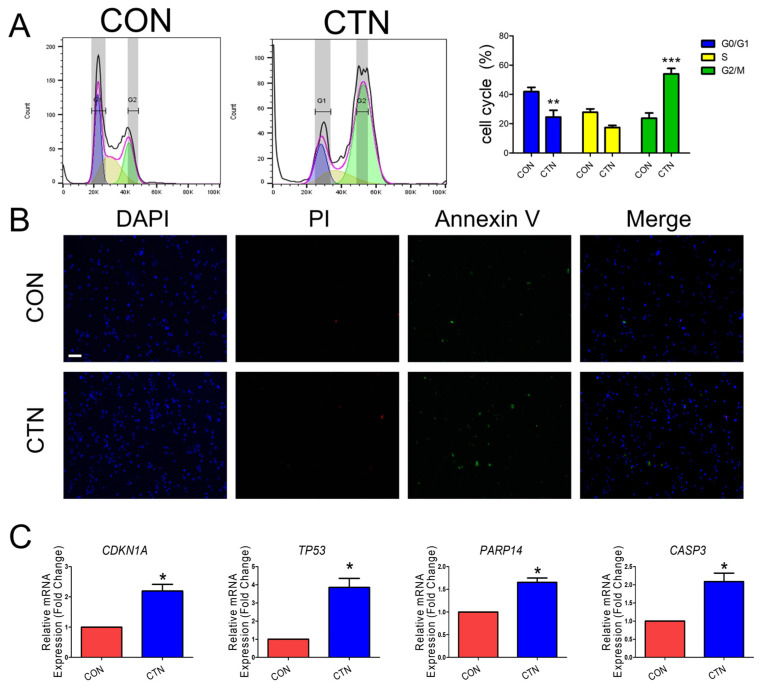
Citrinin induces apoptosis and G2/M phase arrest in porcine intestinal epithelial cells. (**A**) Flow cytometry diagrams showing that IPEC-J2 cells treated with CTN exhibit cell cycle arrest in the G2/M phase compared to untreated cells (CON). (**B**) Fluorescence images of IPEC-J2 cells treated with CTN, stained with propidium iodide (PI; red) and annexin-V (green), including merged images (scale bar = 100 μm). Nuclei were stained with DAPI (blue). (**C**) The mRNA levels of cell cycle arrest-related genes CDKN1A and TP53, as well as apoptosis-related genes CASP3 and PARP14, in the CTN treatment group compared to the control group (*n* = 3). Error bars indicate the standard errors (SEs) of triplicate analyses. Asterisks indicate statistical significance (* *p* < 0.05, ** *p* < 0.01, *** *p* < 0.001).

To investigate the potential association between CTN-induced growth inhibition and cell cycle regulation based on GO data, we conducted a cell cycle distribution analysis using flow cytometry. The analysis showed that the proportion of cells in the G2/M phase was higher in CTN-treated cells than in the control group, while the proportion of cells in the G0/G1 phase was lower (Figure 3A). We performed dual staining using annexin-V and PI, which showed significantly increased apoptosis in treated cells than in untreated cells (Figure 3B). Furthermore, we validated that the mRNA expression levels of the cell cycle-related genes TP53 and CDKN1A, as well as the apoptosis-related genes CASP3 and PARP14, were significantly higher in the treated group compared to the untreated control group (Figure 3C).

### 3.4. CTN Induces Apoptosis and G2/M Phase Cell Cycle Arrest Through the TGF-Beta Signaling Pathway

**Figure 4:**
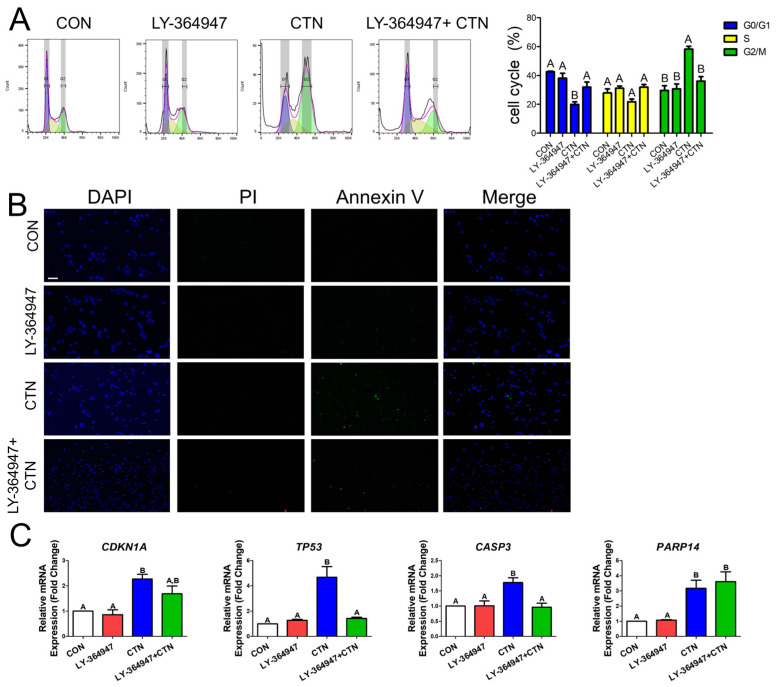
Citrinin induced apoptosis and G2/M phase arrest, and inhibition of the TGF-beta signaling pathway suppressed cell cycle arrest in IPEC-J2 cells. (**A**) Flow cytometry diagrams for IPEC-J2 cells treated with LY-364947, CTN, or both (LY-364947 + CTN) for 24 h, demonstrating how LY-364947, a TGF-beta inhibitor, can alleviate CTN-induced cell cycle arrest. (**B**) Fluorescence images of IPEC-J2 cells stained with propidium iodide (PI; red) and annexin-V (green), including merged images (scale bar = 100 μm). Cells were treated with LY-364947, CTN, or LY-364947 + CTN for 24 h. Nuclei were stained with DAPI (blue). (**C**) The mRNA levels of cell cycle arrest-related genes CDKN1A and TP53, as well as apoptosis-related genes CASP3 and PARP14, in IPEC-J2 cells treated with LY-364947, CTN, or LY-364947 + CTN for 24 h (*n* = 3). Error bars indicate the standard errors (SEs) of triplicate analyses, and the letters above the bars indicate significance groupings (means with the same letter are not significantly different).

To investigate whether CTN-induced growth inhibition is linked to cell cycle regulation and its potential association with the TGF-β signaling pathway, we performed a cell cycle distribution analysis using flow cytometry. (Figure 4A). CTN led to an accumulation of cells in the G2/M phase, increasing from 29.56% in the control to 58.28%, and a decrease in the G0/G1 phase population, dropping from 42.55% to 19.95%. Conversely, when IPEC-J2 cells were cotreated with CTN and LY-364947 for 24 h, no significant changes in cell cycle distribution were observed, with G2/M phase cells at 30.72% and G0/G1 cells at 38.12%. LY-364947 alone had no significant effect. These findings suggest that CTN-induced effects on the cell cycle were alleviated when TGF-beta was inhibited. To determine whether CTN induces apoptosis through TGF-beta signaling, IPEC-J2 cells were treated with CTN, LY-364947, or both, followed by dual staining using annexin-V and PI (Figure 4B). When cells were cotreated with CTN and LY-364947, apoptosis was significantly lower than in cells treated with CTN alone. We examined the mRNA expression levels of cell cycle-related genes, including TP53 and CDKN1A, and found no significant difference in CDKN1A expression between the group cotreated with both CTN and LY-364947 and the CTN-treated group (Figure 4C). However, TP53 expression showed a significant reduction in the cotreated group. Additionally, we investigated the mRNA expression levels of apoptosis-related genes, including CASP3 and PARP14. CASP3 expressions significantly decreased in the group treated with both CTN and LY-364947 compared to the CTN-treated group, whereas PARP14 expression showed no significant difference between the co-treatment and CTN-treated groups.

### 3.5. High-Throughput Screening of NPs to Assess Their Ability to Alleviate Citrinin Toxicity

**Figure 5:**
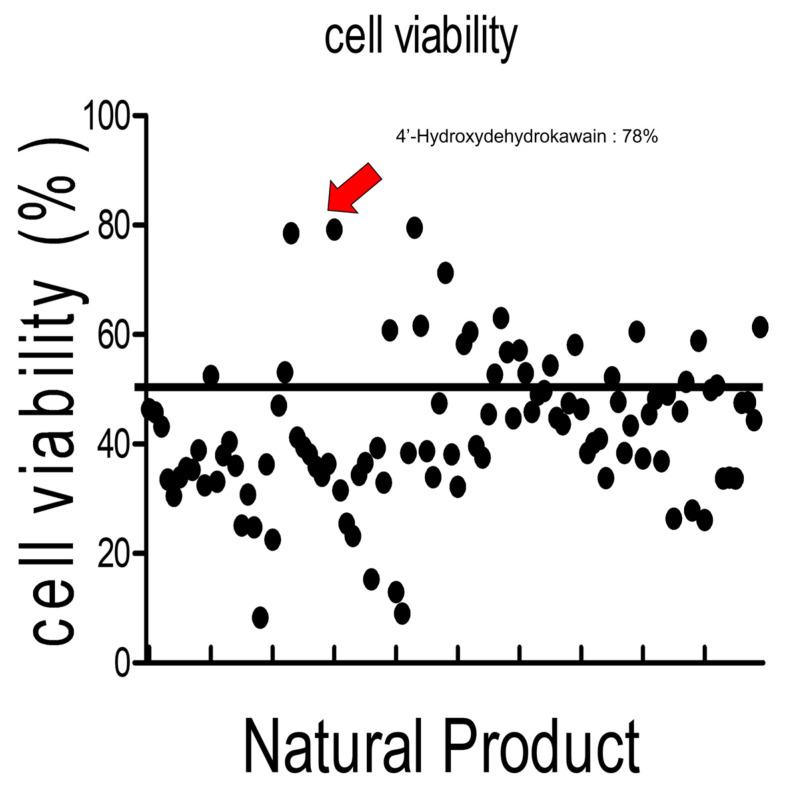
High-throughput screening for natural products mitigates citrinin toxicity. Cell viability was assessed using a WST-1 assay in IPEC-J2 cells treated with CTN at its IC50 value (160 μM) and 2 ng/μL of each of the 100 natural products for 24 h.

We conducted a high-throughput screening to assess whether 100 natural substances could alleviate CTN cytotoxicity (Figure 5). IPEC-J2 cells were exposed to citrinin in the presence of these 100 natural substances. Among the top three substances showing high cell viability, we selected 4-HDK (Figure S3). To further investigate the ability of 4-HDK to alleviate citrinin toxicity, we coadministered CTN and 4-HDK for 24 h.

### 3.6. 4-HDK Mitigates Citrinin-Induced Toxicity by Modulating Gene Expression

**Figure 6:**
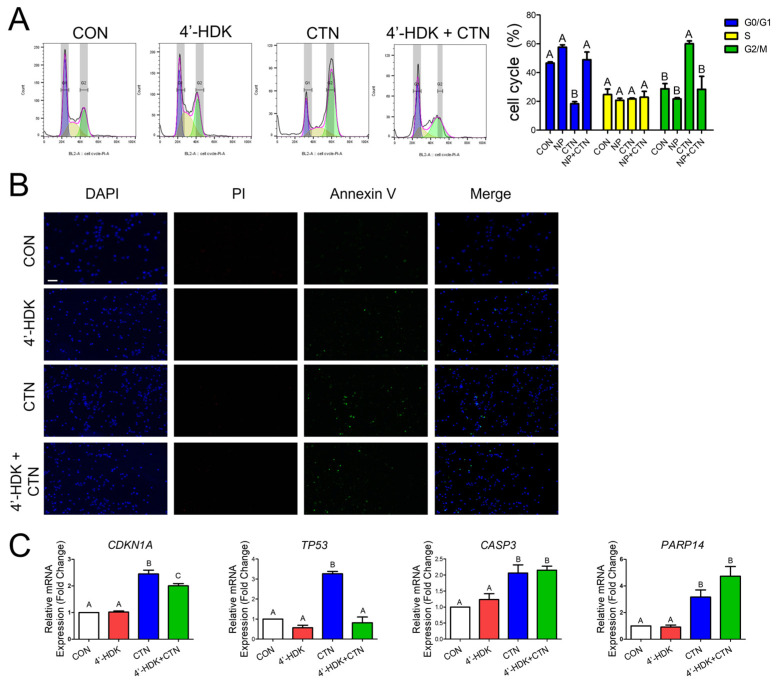
4′-Hydroxydehydrokawain alleviates the toxicity of citrinin. (**A**) Flow cytometry diagrams for IPEC-J2 cells treated with 4-HDK, CTN, or both (4′-HDK+CTD) for 24 h, demonstrating how CTN-induced cell cycle arrest can be alleviated by 4-HDK. (**B**) Images of IPEC-J2 cells stained with propidium iodide (PI; red) and annexin-V (green), including merged staining images (scale bar = 100 μm). Cells were treated with 4-HDK, CTN, or 4′-HDK + CTD for 24 h. Cell staining was performed through single cell staining. Nuclei were stained with 4′,6-diamidino-2-phenylindole (DAPI; blue). (**C**) The mRNA levels of the cell cycle arrest-related genes CDKN1A and TP53 in IPEC-J2 cells treated with 4-HDK, CTN, or 4′-HDK + CTD for 24 h. The expression levels of the cell death-related genes CASP3 and PARP14 mRNA in IPEC-J2 cells treated with 4-HDK, CTN, or 4′-HDK + CTN for 24 h are shown in the panels. (**C**) The error bars represent the standard errors (SEs) of triplicate analyses, and the letters above the bars indicate significance groupings means with the same letter are not significantly different.

A cell cycle distribution analysis was performed using flow cytometry (Figure 6A) on IPEC-J2 cells treated with CTN, 4-HDK at, or both for 24 h. Compared to the untreated cells, the CTN-treated cultures showed an accumulation of cells in the G2/M phase and a decrease in the G0/G1 phase population. However, when IPEC-J2 cells were cotreated with CTN and 4-HDK, the effects of CTN were alleviated. Specifically, the addition of 4-HDK to the CTN treatment increased the proportion of cells in the G0/G1 phase from 18.28 to 48.88% and decreased the proportion of cells in the G2/M phase from 60.04 to 28.31%. The 4-HDK treatment alone had no significant effect on the cell cycle distribution. Additionally, we investigated the mRNA expression levels of cell cycle-related genes TP53 and CDKN1A (Figure 6B). For CDKN1A, the group cotreated with CTN and 4-HDK showed a significant increase in expression compared to the untreated group but a significant decrease compared to the CTN-treated group. For TP53, expression in the cotreated cells was significantly and noticeably lower than in the CTN-treated cells. To assess whether 4-HDK alleviates CTN-induced cell death, dual staining with annexin-V and PI was performed (Figure 6C). The 4-HDK group showed results similar to those of the untreated group, and the CTN treatment induced observable cell death. However, when CTN and 4-HDK were coadministered, cell death was significantly lower than when treated with CTN alone. Additionally, we investigated the mRNA expression levels of cell cycle-related genes TP53 and CDKN1A (Figure 6C). For CDKN1A, the group cotreated with CTN and 4-HDK showed a significant increase in expression compared to the untreated group but a significant decrease compared to the CTN-treated group. For TP53, expression in the cotreated cells was significantly lower than in the CTN-treated cells. We also examined the mRNA expression levels of cell death-related genes CASP3 and PARP14, finding no significant differences between the group cotreated with both CTN and 4-HDK and the CTN-treated group.

## 4. Discussion

In animals, CTN is produced by various fungi, including the genera Penicillium, Aspergillus, and Monascus, during the cultivation, harvesting, storage, and transportation of grains, and it has also been detected in processed foods such as fruits, vegetable and fruit juices, medicinal and aromatic herbs, and moldy cheeses [30,31]. Citrinin is a mycotoxin with genotoxic and nephrotoxic properties, presenting concerns for both food safety and environmental health [32]. When ingested through mycotoxin-contaminated feed, it is absorbed and accumulates in the intestinal epithelium [33]. Concentrated CTN disrupts tight junctions, compromising epithelial barrier integrity by impairing cell proliferation, inhibiting stem cell renewal, and suppressing the regeneration cycle of the intestinal epithelium [34]. According to a study, in pigs, a CTN concentration of 80 μM has been reported to inhibit oocyte maturation in vitro [35]. In humans, chromosomal abnormalities have been observed in primary human renal proximal tubular epithelial cells at CTN concentrations of 10–20 μM [36]. Although the risks of CTN are well known, studies on its molecular mechanisms in pig intestinal epithelial cells remain limited.

Therefore, we conducted gene expression profiling in IPEC-J2 cells after CTN treatment to determine whether CTN induces cell cycle arrest through TGF-β signaling. The molecular mechanisms of CTN activity in IPEC-J2 cells require additional research. We profiled the expression of 1379 DEGs in porcine intestinal epithelial cells, and the “regulation of the cell cycle” was identified as the prominent GO term, and the cellular senescence, cell cycle, and TGF-beta signaling pathways were identified as the primary pathways. The TGF-beta signaling pathway is known to be involved in cell aging and the cell cycle [37]. Based on these findings, we hypothesized that CTN induces cell cycle arrest through the TGF-beta signaling pathway and conducted experiments to test this hypothesis. The cell cycle is regulated by a series of complexes formed by cyclins and cyclin-dependent kinases (CDKs) [38]. Cell cycle progression is positively driven by these cyclin–CDK complexes, which operate through multiple processes, including cyclin and CDK subunit assembly, activating and inhibitory phosphorylation and dephosphorylation events, and interactions with cyclin-dependent kinase inhibitors CKIs [39]. On the other hand, CKIs negatively regulate cell cycle progression [40]. The p53-p21-RB pathway governs the expression of a wide array of genes, including many that play pivotal roles in regulating the cell cycle [41]. Consequently, the overexpression of p53 or p21 causes incorrect regulation and suspension of the cell cycle.

To confirm CTN-induced cell cycle arrest in the small intestine, flow cytometry and real-time PCR analysis of the expression of the cell cycle arrest-related genes TP53 and CDKN1A were conducted [24,42]. The flow cytometry analysis revealed an increase in the number of cells in the G2/M phase. Furthermore, to confirm CTN-induced cell apoptosis in the small intestine, annexin-V and PI staining and real-time PCR analysis of the cell apoptosis-related genes *CASP3* and *PARP14* [43,44] were conducted. The annexin-V and PI staining revealed the occurrence of apoptosis. After showing the induction of the TGF-beta signaling pathway by CTN, we confirmed that this was the mechanism of cell cycle arrest and apoptosis by simultaneously treating cells with CTN and the TGF-beta inhibitor LY-364947. Following this treatment, flow cytometry analysis revealed that the proportion of cells in the G2/M phase was lower in the group treated with both the LY-364947 and CTN than in the group treated with CTN alone. Furthermore, annexin-V and PI staining indicated that LY-364947 reduced apoptosis in CTN-treated cells. This evidence suggests that CTN can indirectly induce apoptosis and cell cycle arrest in IPEC-J2cells through the TGF-beta signaling pathway.

One hundred natural products, including 4-HDK, an AKH extract provided by the National Academy of Oriental Medicine, were screened using a high-throughput screening method. The 4-HDK treatment alleviated the cytotoxicity of citrinin. Subsequently, experiments were conducted to further investigate the effects of 4-HDK on citrinin. Flow cytometry analysis revealed that the proportion of cells in the G2/M phase was lower in the group treated with both 4-HDK and CTN than in the group treated with CTN alone. Additionally, annexin-V and PI staining demonstrated a decrease in cell death when 4-HDK was combined with the CTN treatment. This indicates that, like LY-364947, 4-HDK can inhibit the toxicity of citrinin, alleviating cell cycle arrest and cell death in porcine intestinal epithelial cells. However, our current data do not provide direct evidence of HDK binding to a representative site to confirm its role as a direct repressor. Future studies will aim to identify the specific binding sites and confirm the direct interaction between HDK and its molecular targets.

Through this study, we demonstrated that 4-HDK can mitigate the cytotoxicity of CTN in IPEC-J2 cells. This suggests the potential of 4-HDK to prevent the destruction of intestinal epithelial cells induced by CTN. As a result, we hypothesize that 4-HDK may inhibit the absorption of CTN in the intestines and consequently reduce its accumulation in meat. However, these results were obtained in vitro, and we cannot guarantee that the same outcomes will be observed in vivo. Furthermore, it remains uncertain whether similar effects will be seen in animals other than pigs, such as humans or cattle. To confirm this, further research is essential.

## 5. Conclusions

**Figure 7:**
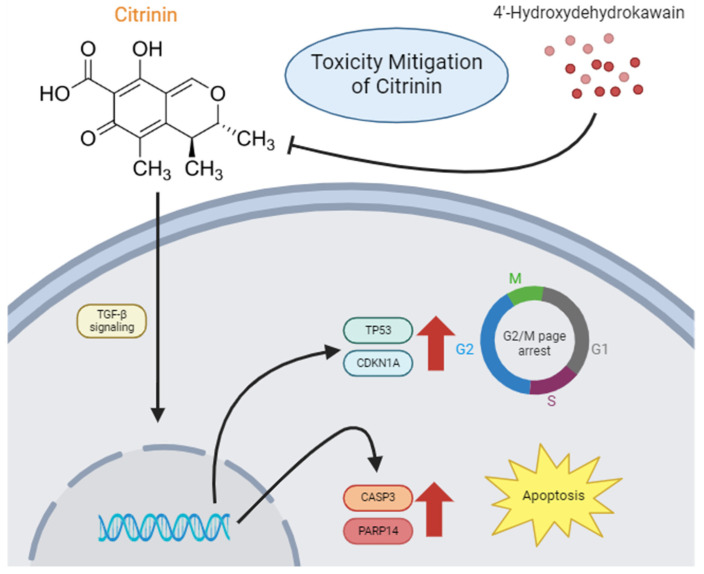
CTN induces apoptosis and cell cycle arrest via the TGF-β signaling pathway, and 4-HDK mitigates these effects. According to this hypothesis, CTN activates TGF-β signaling to express CDKN1A and TP53 genes. Arrows pointing to cell cycle arrest and apoptosis indicate the expression of the genes involved.

This study provides compelling evidence that CTN induces apoptosis and G2/M phase cell cycle arrest in IPEC-J2 cells through the activation of the TGF-β signaling pathway. Specifically, our results indicate that CTN upregulates TP53 and CDKN1A expression, leading to increased apoptosis and cell cycle disruption. The use of the TGF-β receptor inhibitor LY-364947 confirmed that CTN-induced toxicity is mediated through this pathway. Importantly, we observed that 4-HDK improved cell viability and reduced apoptosis in CTN-treated cells; however, its specific mechanism of action remains to be determined. While our study establishes a strong link between TGF-β activation and CTN-induced toxicity, further investigation is needed to elucidate whether 4-HDK directly modulates this pathway or exerts its protective effects through alternative mechanisms. Specifically, assessing the phosphorylation status of SMAD2/3 and other downstream effectors would provide deeper insights into the mechanistic role of TGF-β signaling in CTN toxicity. Future research should also explore additional pathways influenced by 4-HDK to confirm its role in mitigating mycotoxin-induced damage. Overall, our findings contribute to the understanding of CTN toxicity mechanisms and propose 4-HDK as a promising natural intervention for protecting intestinal epithelial cells from mycotoxin-induced stress in livestock (Figure 7).

## Figures and Tables

**Table 1:** List of PCR primers.

Genes	Description	Accession No.	Sequence (5’-3’)	
TGM2	Transglutaminase 2	XM_003359989.5	Forward	CGC CTT CTC TCC GTA TGA CT
			Reverse	TTT TGT GCT TCT TCC TGT GC
BEX5	Brain expressed X-linked 5	XM_005657871.3	Forward	TAT CCT CAG CAG GTC CAC GT
			Reverse	CTT CTT CATVTCC GCA TTT GA
FOXJ1	Forkhead box protein J1	XM_003357959.4	Forward	CTG TCC TCC CCA GGT CTC TA
			Reverse	AAA TCT CCT TGC TCC ACC AG
BCAS1	Brain-enriched myelin- associated protein 1	NM_001110175.1	Forward	GCC CCC GAC AGA GAA TAA TA
			Reverse	CAC TTG AGC ATC CAA CAT CG
CASP3	Caspase3	NM_214131	Forward	CTC AGG GAG ACC TTC ACA AC
			Reverse	GCA CGC AAA TAA AAC TGC TC
PARP14	Poly (ADP-ribose) polymerase family member 14	XM_021070260.1	Forward	CCA CTC TCT GTG TTC CCG TA
			Reverse	GGT GAG AGA CAC AAG GGC AT
CDKN1A	Cyclin-dependent kinase inhibitor 1A	XM_013977858.2	Forward	GGT TCC CCA GTT CTA CCT CA
			Reverse	GCG TCT CGC TTC ATC ATT TA
TP53	Transformation-related protein 53	NM_213824.3	Forward	TGC TGT TTC CGT GTG TTT TT
			Reverse	ATG GGG AGG GAG GTT ATC A
GABDH	Glyceraldehyde-3- phosphate dehydrogenase	NM_001206359	Forward	ACA CCG AGC ATC TCC TGA CT
			Reverse	GAC GAG GCA GGT CTC CCT AA

## Data Availability

Data are contained within the article.

## Supplementary Materials

The following supporting information can be downloaded at https://www.mdpi.com/article/10.3390/toxics13040315/s1, Figure S1: GO and KEGG of upregulated differentially expressed genes, Figure S2: GO and KEGG of downregulated differentially expressed genes, Figure S3: Cell viability analysis of the top 3 natural products with high alleviate citrinin toxicity as a result of high-throughput screening.

## Abbreviations

The following abbreviations are used in this manuscript:

CTN	Citrinin
IPEC-J2	Intestinal Porcine Epithelial Cells-J2
TGF-β	Transforming Growth Factor-beta
4-HDK	4′-Hydroxydehydrokawain
PI	Propidium iodide
DAPI	4′,6-Diamidino-2-phenylindole
RT-qPCR	Reverse Transcription Quantitative Polymerase Chain Reaction
DEG	Differentially expressed gene
GO	Gene Ontology
KEGG	Kyoto Encyclopedia of Genes and Genomes
FACS	Fluorescence-Activated Cell Sorting
NP	Natural product
