# Supplementary material for: 4′-Hydroxydehydrokawain Mitigate the Cytotoxicity of Citrinin in Porcine Intestinal Epithelial Cells
Source: Toxics. 2025 Apr 18;13(4):315. doi: 10.3390/toxics13040315 (PMC12031180; doi:10.3390/toxics13040315)
Supplement: Supplementary file 1 [file toxics-13-00315-s001.zip › toxics-3543677-supplementary.pdf]

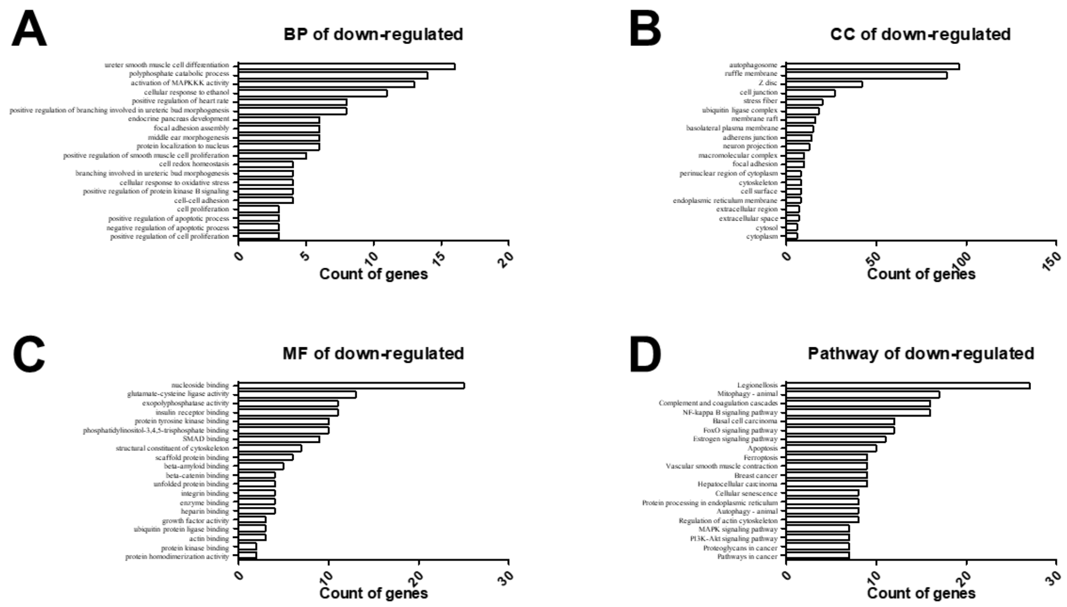

Figure S1: GO and KEGG of upregulated differentially expressed genes

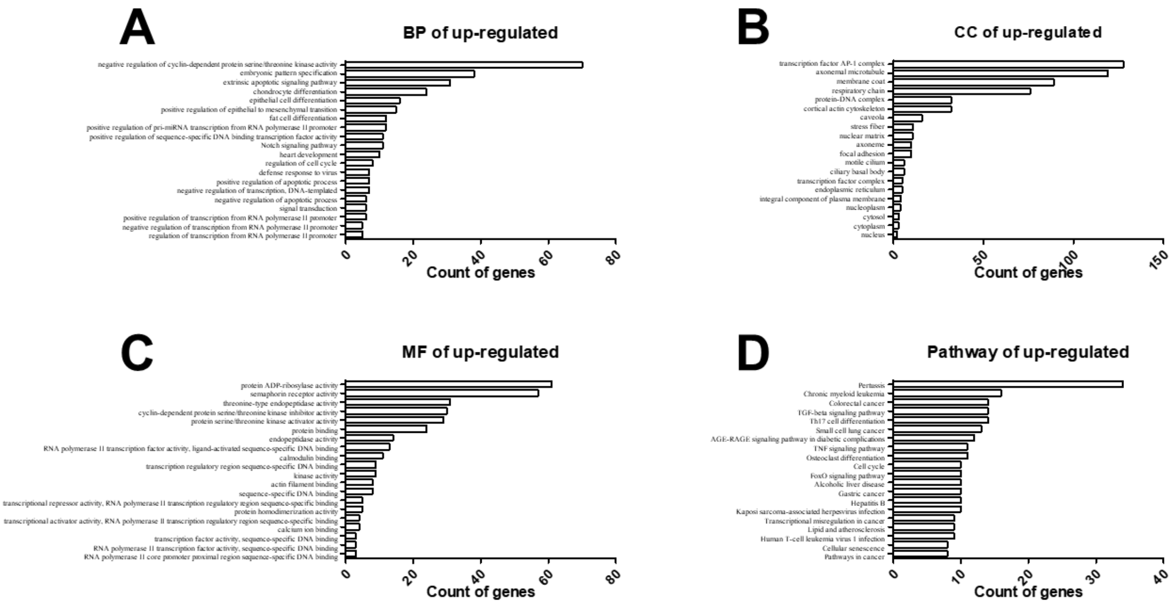

Figure S2: GO and KEGG of downregulated differentially expressed genes

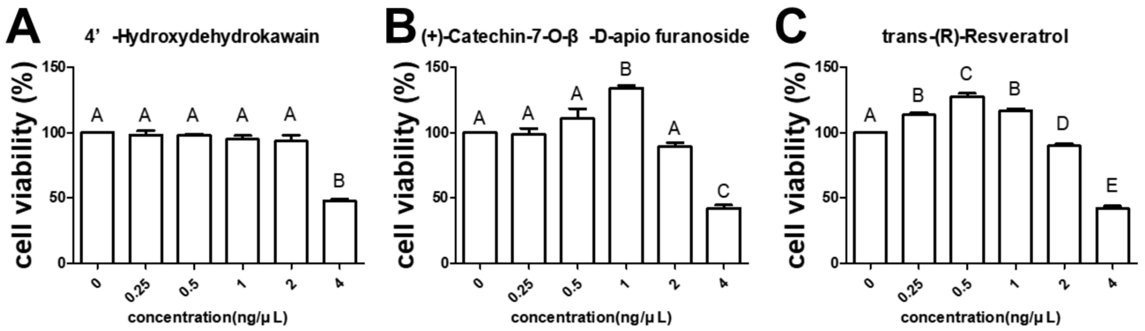

Figure S3: Cell viability analysis of the top 3 natural products with high alleviate citrinin toxicity as a result of high throughput screening.
